# Abrogation of Endogenous Glycolipid Antigen Presentation on Myelin-Laden Macrophages by D-Sphingosine Ameliorates the Pathogenesis of Experimental Autoimmune Encephalomyelitis

**DOI:** 10.3389/fimmu.2019.00404

**Published:** 2019-03-19

**Authors:** Yi Ban, Wenjuan Dong, Lixing Zhang, Tian Zhou, Ahmad S. Altiti, Khaleel Ali, David R. Mootoo, Victoria A. Blaho, Timothy Hla, Yi Ren, Xiaojing Ma

**Affiliations:** ^1^Department of Microbiology and Immunology, Weill Cornell Medicine, New York, NY, United States; ^2^State Key Laboratory of Microbial Metabolism, Sheng Yushou Center of Cell Biology, Shanghai Jiaotong University, Shanghai, China; ^3^Department of Biomedical Sciences, College of Medicine, Florida State University, Tallahassee, FL, United States; ^4^Key Laboratory of Biorheological Science and Technology, College of Bioengineering, Chongqing University, Chongqing, China; ^5^Department of Chemistry, Hunter College, City University of New York, New York, NY, United States; ^6^Department of Pathology and Laboratory Medicine, Center for Vascular Biology, Weill Medical Medicine, New York, NY, United States; ^7^Sanford Burnham Prepys Medical Discovery Institute, La Jolla, CA, United States

**Keywords:** endogenous myelin-derived galcer, antigen presentation, IL-17, EAE progression, D-sphingosine

## Abstract

**Background:** Although myelin is composed of mostly lipids, the pathological role of myelin lipids in demyelinating diseases remains elusive. The principal lipid of the myelin sheath is β-galactosylceramide (β-Galcer). Its α-anomer (α-Galcer) has been demonstrated to be antigenically presented by macrophages via CD1d, a MHC class I-like molecule. Myelin, which is mostly composed of β-Galcer, has been long considered as an immunologically-inert neuron insulator, because the antigen-binding cleft of CD1d is highly α-form-restricted.

**Results:** Here, we report that CD1d-mediated antigenic presentation of myelin-derived galactosylceramide (Mye-GalCer) by macrophages contributed significantly to the progression of experimental autoimmune encephalomyelitis (EAE). Surprisingly, this presentation was recognizable by α-Galcer:CD1d-specific antibody (clone L363), but incapable of triggering expansion of *i*NKT cells and production of *i*NKT signature cytokines (IFNγ and IL-4). Likewise, a synthesized analog of Mye-Galcer, fluorinated α-C-GalCer (AA2), while being efficiently presented via CD1d on macrophages, failed to stimulate production of IFNγ and IL-4. However, AA2 significantly exacerbated EAE progression. Further analyses revealed that the antigenic presentations of both Mye-GalCer and its analog (AA2) in α-form via CD1d promoted IL-17 production from T cells, leading to elevated levels of IL-17 in EAE spinal cords and sera. The IL-17 neutralizing antibody significantly reduced the severity of EAE symptoms in AA2-treated mice. Furthermore, D-sphingosine, a lipid possessing the same hydrophobic base as ceramide but without a carbohydrate residue, efficiently blocked this glycolipid antigen presentation both *in vitro* and in spinal cords of EAE mice, and significantly decreased IL-17 and ameliorated the pathological symptoms.

**Conclusion:** Our findings reveal a novel pathway from the presentation of Mye-GalCer to IL-17 production, and highlight the promising therapeutic potential of D-sphingosine for the human disorder of multiple sclerosis.

## Introduction

Experimental autoimmune encephalomyelitis (EAE) is an established mouse model that shares clinical and pathological features of human multiple sclerosis (MS), an inflammatory demyelinating disease of the central nervous system (CNS). Various myelin-based proteins, such as myelin basic protein, proteolipid protein, and myelin oligodendroglial glycoprotein (MOG), have been proposed as the origin to the encephalitogenic peptides presented to T cells via the MHC complexes, and have been one of the foci of the studies in the field. However, the immunological role of the principal lipid of myelin sheath, galactosylceramide (GalCer), in EAE is not clear. Both protective and aggravating effects of GalCer and its analogs have been reported (1–3).

Glycolipid antigen presentation via CD1 has been shown to activate CD1-restricted T cells (4). The CD1 molecule structurally mimics MHC class I with a highly similar tertiary fold. However, the antigen-binding cleft of CD1 is specific for lipids. Crystal structure analyses of human and mouse CD1d (a CD1 subtype) and α-galactosylceramide (α-GalCer, a glycolipid in a derivative of a glycolipid from the marine sponge *Agelas mauritianus*) have revealed that the sphingosine and the longer alkyl chain of the ligand fit into two deep hydrophobic pockets, whereas the galactose forms hydrogen-bonds with the outer portion of CD1d. Such an orientation of amphipathic lipids allows hydrophilic carbohydrate residues of the lipid to interact with the TCR for activating immune responses (5).

Ceramides linked to β-galactose, rather than α-galactose, are widely occurring in mammalian nervous tissues (e.g., myelin). It was believed that α-GalCer was absent, because only one UDP-galactose:ceramide β_1→4_ galactosyl transferase was found in mammalian genomes (6). However, recent studies using α-Galcer: CD1d specific antibody L363 detected a trace amount (0.02%) of α-Galcer in mammalian species as an immune agonist to invariant NKT cells (iNKT) (7–9). Additionally. It has still been long ignored that anomerization (β- ↔ α-anomer conversion) could occur in acidic condition, such as in lysosome and late endosome of macrophages. Therefore, positive detection of mammalian cells by α-GalCer:CD1d specific antibody L363 (7–9) is possible, and suggests two possibilities: (1) selective presentation of trace amount of α-GalCer or (2) loading of anomerized β → α-GalCer onto CD1d in lysosome/late endosome.

Imbalanced cytokine milieu also plays an important role in initiation and perpetuation of the autoimmune pathology. Increased production of pro-inflammatory cytokines (e.g., TNFα, IFNγ, and IL-17) occurs in cerebrospinal fluids, blood mononuclear cells and brain lesions in MS patients during an exacerbation phase (10, 11); whereas immune-suppressing cytokines (e.g., IL-4 and IL-10) are elevated during disease remission (12). Interestingly, activated NKT cells are capable of producing both pro- and anti-inflammatory cytokines described above (13). Indeed, contradictory effects of iNKT cells have also been reported with a consensus that timing, administration routes and extent of iNKT activation all determine its role, protective or potentiating, in EAE (2, 14, 15). Therefore, a more well-defined protective mechanism is needed for developing therapeutic strategies for MS patients.

Variations in glycolipid structures impact on their interaction with CD1d and NKT cells, and in turn on cytokine production of NKT cells (16). Herein, we identified induction of IL-17 by myelin-derived GalCer (Mye-Galcer). Briefly, endogenous Mye-Galcer was found presented on myelin-laden macrophages. This antigenic presentation of glycolipid was repeatedly recognized by antibody L363, which is highly specific to the α-Galcer:CD1d complex (7). To dissect the role of Mye-GalCer from other components in myelin, we designed and synthesized an α-galactosyl lipid that resembles the Mye-GalCer in terms of its antigenic presentation and downstream cytokine production. Both Mye-Galcer and its synthetic analog induced IL-17 production. We also observed that D-sphingosine, a myelin lipid without a carbohydrate residue, efficiently blocks the presentation of Mye-GlyCer. MOG-induced EAE mice treated with D-sphingosine exhibit much milder disease severity and significantly improved survival rates. Our study shed a considerable light on the therapeutic potentials of D-sphingosine for MS patients.

## Materials and Methods

### Mice

WT C57BL/6 mice were purchased from the Jackson Laboratories (Bar Harbor, ME, USA). All mice were maintained in a pathogen-free facility. All animal protocols are in compliance with the Guide for the Care and Use of Laboratory Animals (National Institutes of Health) and proved by the Research Animal Resource Center at Weill Cornell Medicine.

### EAE Induction and Clinical Scoring

Female C57BL/6 mice (12 weeks of age or elder) were immunized subcutaneously with 200 μg/mouse MOG_35−55_ peptide (Alpha Diagnostic Intl. Inc) emulsified in a 1:1 ratio with complete Freund's adjuvant (CFA, 2 mg/ml heat-inactivated mycobacterium tuberculosis). Two doses of 200 ng pertussis toxin were injected intraperitoneally on Day 1 (immunization day) and Day 3. Severity of disease symptoms were scored from 0 to 5 in an increasing order of severity: 0, normal; 1, limp tail or hind limb weakness; 2, limp tail and limb weakness or weakness of 2 or more limbs; 3, severe limb weakness or single limb paralysis; 4, paralysis of 2 limbs; 5, moribund or death. For evaluating the exacerbating effects of AA2 in EAE progression, the 2nd dose of PTX was waived for both control and AA2-treated EAE mice.

### Glycolipid

α-C-GalCer was prepared as previously described (17). The new synthetic glycolipid AA2 was synthesized from a known precursor following protecting group and amidation procedures as described previously (18). Galcer purified from bovine spinal cord and D-sphingosine were purchased from Matreya LLC and Sigma-Aldrich respectively.

### Mouse Treatment

Glycolipid AA2 and D-sphingosine were initially dissolved in DMSO, followed by dilution with PBS and brief sonication. After EAE induction, mice were injected i.p. every other day with 10 μg AA2/mouse or with vehicle. Daily injection of D-spingosine via i.p. or i.v. started on day 3 after EAE induction. Anti-IL-17 neutralizing antibody (100 μg/mouse, Biolegend) or its isotype control (Rat IgG1) was given at Day 3, 5, 7, 9, and 11 after EAE induction. All EAE mice were euthanized on day 20–25. Spinal cords and brains were fixed by 4% paraformaldehyde (PFA) for immunohistochemistry (IHC) and immunofluorescence staining.

### Cells Culture and *in vitro* Treatment

*In vitro* macrophages culture: Bone marrow aspirates of C57BL/6 mice were cultured in DMEM supplied with 10% FBS, 2 mM glutamine, penicillin and 20% L929 cells supernatant for 6–7 days until mature mouse primary macrophages were formed. The macrophages were treated with myelin debris or synthetic glycolipids for 12, 24, and 48 h before examination of their molecular traits. Myelin debris were prepared as described previously (19). Untouched T cells-enriched splenetic cells were acquired by depleting whole spleen single cell suspension of B cells via B220 microbeads (Myltenyi Biotec) through LD columns (Myltenyi Biotec) according to the manufacturer's manual. T cell percentage (85–92%) and viability (≥95%) were verified by flow cytometric analysis. The T cells enriched splenetic cells were added to macrophage layer to form the macrophage-T cell co-culture system. After 2–3 h initial co-culture, myelin debris, glycolipids, or D-sphingosine were further added to the co-culture system.

### Capture Antibody-Coated Beads (CABs) Assays

Briefly, mouse IL-17A capture antibody-coated beads were added to supernatants according to instructions of the manufacturer (BD Bioscience). After incubation and 3 washes, the detection antibody (anti-mouse IL-17A-PE) was added, followed by flow-cytometric analysis.

### Flow Cytometry Analysis

Antibodies employed in flow cytometric analysis were obtained from various commercial sources: anti-CD1d (Biolegend, 1B1), anti-αGalcer:CD1d (eBioscience, L363), CD86 (BD Bioscience, GL1), TCRαβ (eBioscience,IP26), F4/80 (eBioscience, BM8), IL17-A (Biolegend,TC11-18H10.1), NK1.1 (Biolegend, PK136), and CD3 (Biolegend, 17A2). Cells were blocked with anti-CD16/32 antibody (Biolegend) for 15 min before incubation with fluorescently labeled antibodies at a concentration of 2 μg/ml for 45 min on ice. Stained cells were washed once with FACS buffer and analyzed by FACSan (Becton Dickinson). For internal staining, cells stained with surface makers were fixed in 2% PFA Fixation buffer (eBioscience) at 4°C overnight, followed by 3 washes with permeabilization buffer (R&D) and incubation with permeabilization buffer for 20 min on ice before staining with internal antibody. Stained cells were washed once with permeabilization buffer and suspended in PBS for analysis. Flow cytometry data were processed with FlowJo software (Tree Star, inc.,).

### ELISA

Cell supernatant and mice serum were collected and stored at −80°C until analysis. Spinal cords were excised and homogenized via sonication with PBS supplemented with protease inhibitor cocktail (Roche). Spinal homogenates were centrifuged, and supernatants were collected and stored at −80°C until analysis. IFNγ, IL-4, and IL-17A were measured with ELISA kit purchased from eBioscience. The experiments were performed according to the manufacturer's instruction. Briefly, the plates were coated with capture antibody at 4°C overnight. The coated plate were blocked with assay diluent for 1 h at room temperature (RT). Samples were added to the plate and incubated at RT for 2 h. Avidin-HRP and TMB substrate were employed for detection of the cytokine signal of interest. After the reactions were stopped with stop solution, the plates were read for 450 nm values with subtraction of 570 nm values within 30 min.

### *In vitro* Immunofluorescence Staining

Mature macrophages were immobilized on coverslips and treated with myelin debris for various periods of time as indicated. At the end of treatment, cells were washed with PBS, followed by fixation and permeabilization with 4% PFA and 0.2% Triton X100, respectively. The macrophages were further blocked (PBS containing 5% FBS) and stained with aGalcer:CD1d (eBioscience, L363) and adipored (Lonza) overnight at 4°C. After counterstaining with 2.5 μg/ml DAPI (Invitrotgen) for 5 min at RT, cells were mounted and subject to imaging with an inverted fluorescence microscope (OLYMPUS).

### Pathology of EAE Mice

Specimens (spinal cords and brains) were embedded in low-melting-point paraffin wax and cut into 7 μm sections. Sections stained with haematoxylin and eosin (H&E) were microscopically examined for general histopathology assessment. The immunostaining of iba1, IL-17A, and α-GalCer were performed using a standard immunofluorescence protocol. Briefly, formalin-fixed paraffin-embedded sections were dewaxed and rehydrated in xylene, 100% ethanol, 95% ethanol, 70% ethanol, 50% ethanol, and PBS sequentially. Acidic antigen retrieval and 5% sheep serum blocking were performed before incubation with primary antibodies overnight at 4°C. Sections were washed 3 times with washing buffer (0.5% Tween 20 in PBS, pH 7.2), and further subject to incubation with secondary antibody conjugated with indicated fluorescence for 45 min at RT. After counterstaining with Hoechst 33342, sections were mounted and examined by laser scanning microscope FV1000 (OLYMPUS).

### Statistical Analysis

Student's *t*-test was employed to calculate statistical significance for difference between groups. A *P* < 0.05 was considered statistically significant.

## Results

### Molecular Features of Myelin-Laden Macrophages

**Figure 1:**
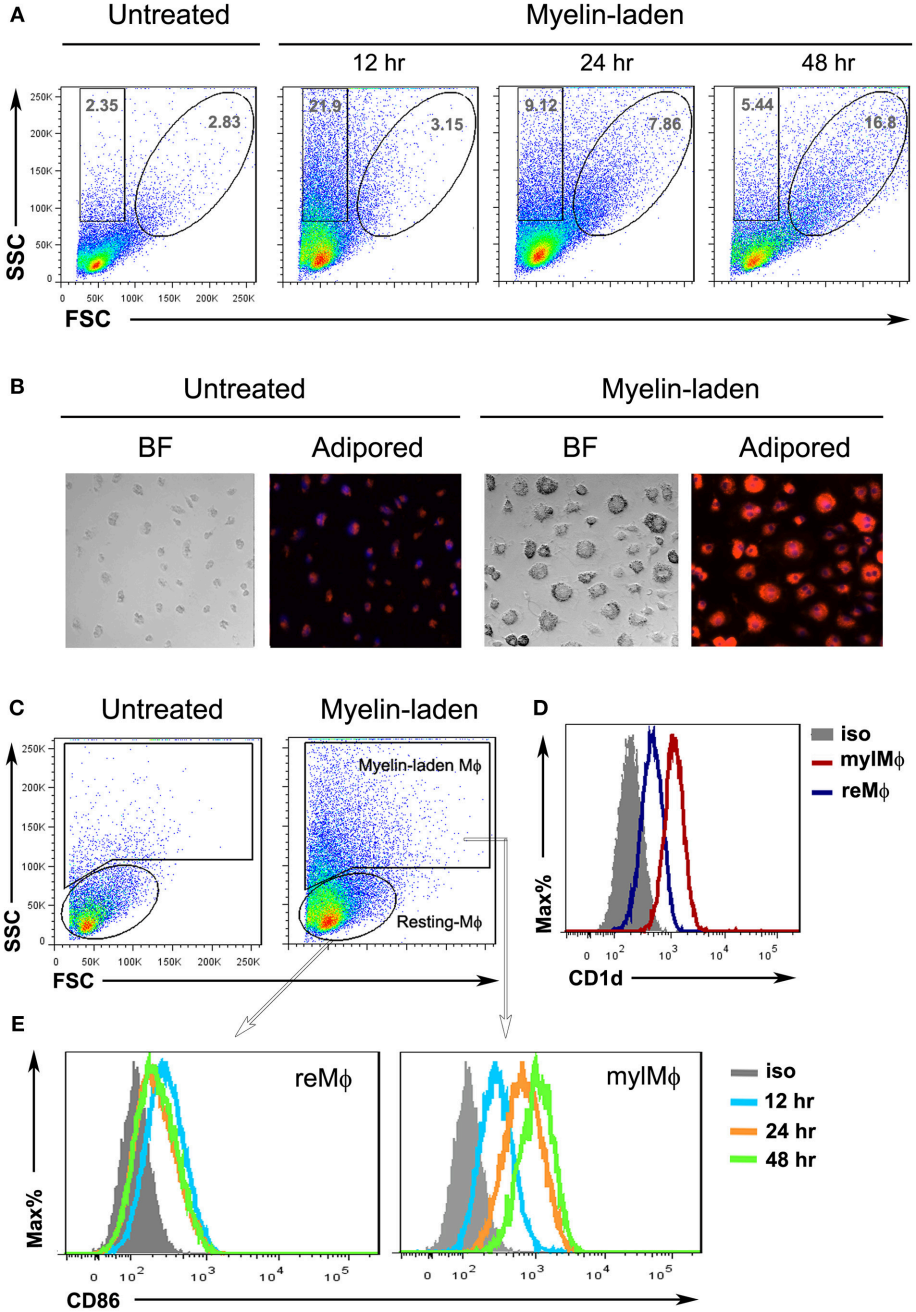
Characterization of myelin-laden macrophages. **(A)** Flow cytometric analysis of resting (reMφ) vs. myelin-laden macrophages (mylMφ) for side- and forward-scatters at indicated time points after myelin internalization. **(B)** Fluorescent microscopy of resting (reMφ) vs. myelin-laden macrophages (mylMφ) 48 h post- myelin-internalization (lipid: red; nucleus: blue; BF: Bright Field; amplification: 20x). **(C)** Gating strategies for resting and myelin-laden macrophages. **(D,E)** Flow cytometric analysis of CD1d expression **(D)** and CD86 expression **(E)** by resting vs. myelin-laden macrophages.

The myelin-targeting autoimmune responses rely on both innate and adaptive components. Macrophages are professional phagocytes that engulf and digest myelin debris. Upon phagocytosis, macrophages serve as a bridge between innate and adaptive immune responses (20). To characterize the myelin-laden macrophages, bone marrow (BM)-derived macrophages were fed with myelin debris and analyzed for their molecular traits. A side scatter (SSC)^high^/forward scatter (FSC)^low^ population, indicating an increased inner complexity of cells, was observed at 12 h after addition of myelin debris. This population became SSC^high^/FSC^high^ at 48 h, suggesting that the myelin-laden macrophages also augmented in size (Figure 1A). Immobilized, myelin-laden macrophages were stained with an orange-to-red lipid dye (adipored), and then subject to microscopic observations. The untreated macrophages appeared to be relatively small, and exhibited low adipored fluorescence; whereas myelin-fed macrophages were found complex, enlarged, and adipored-high (Figure 1B). Since the only lipids in the culture were the purified myelin lipids, the observation of lipid-positive macrophages indicated that the SSC^high^/FSC^high^ population represented myelin-laden macrophages. We further explored the differences between resting macrophages (SSC^low^/FSC^low^) and myelin-laden macrophages (from SSC^high^/FSC^low^ to SSC^high^/FSC^high^) (Figure 1C). The latter showed significant up-regulation of CD1d in comparison to the resting macrophages (Figure 1D). A time-dependent gradual enhancement of the co-stimulatory molecule CD86 on the cell surface (Figure 1E) further suggested that myelin-laden macrophages are poised to activate immune responses.

### Antigenic Presentation of Endogenous Mye-GalCer via CD1d on Myelin-Laden Macrophages

**Figure 2:**
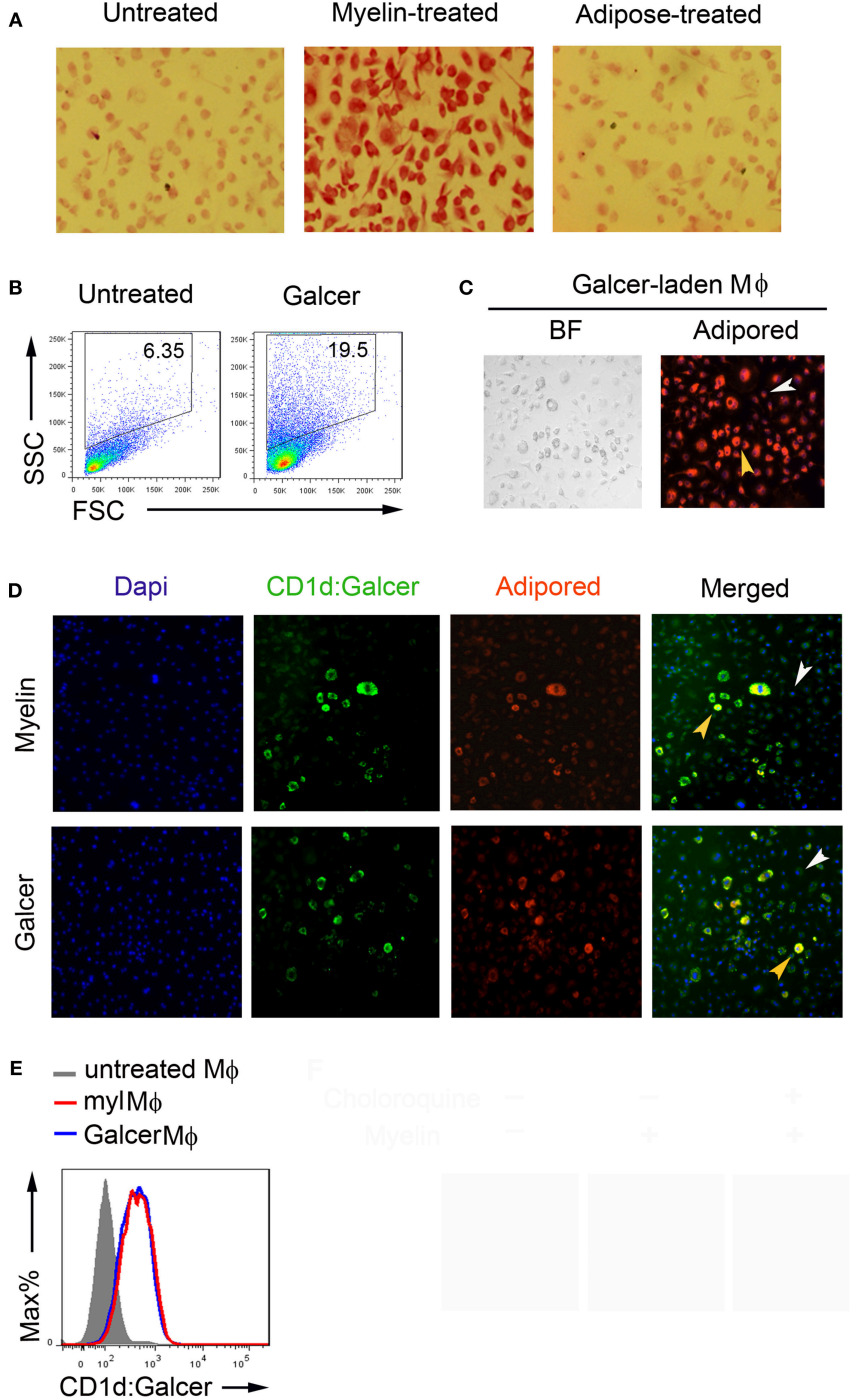
Antigenic presentation of Mye-Galcer by myelin-laden macrophages. **(A)** Periodic Acid-Shiff reactions (magenta) of resting macrophages, myelin-laden macrophages and adipose-treated macrophages. **(B)** Gating strategies for resting and Galcer-laden macrophages. **(C)** Microscopy of Galcer-laden macrophages stained with a lipid dye (Adipored). Control: resting macrophages, indicated by white arrow head; Galcer-laden macrophages indicated by yellow head. BF: Bright Field **(D)** Fluorescent microscopy of antigenic presentation of Galcer on myelin- and Galcer-laden macrophages stained with anti-CD1d:Galcer (L363, green), adipored (red), and Dapi (blue). Resting macrophages and myelin-laden macrophages were indicated by white and yellow arrow head, respectively. **(E)** Flow cytometric analysis of CD1d up-regulation by myelin- and Galcer-laden macrophages.

The discovery that both lipid and glycolipid antigens could be recognized by T cells has greatly broadened our knowledge of antigen repertoire (21). To determine whether the galactose moiety were remained on myelin lipids long enough inside macrophages to be loaded onto CD1d, we stained myelin-laden macrophages with Periodic Acid-Shiff (PAS), which specifically reacts with saccharides to give a magenta color, at 24 h after initial feeding of myelin debris. As shown in Figure 2A, a more eminent magenta color was exhibited by myelin-laden macrophages when compared with non-treated or adipose homogenate-fed macrophages. It suggested that at least a portion of myelin lipids were intact as glycolipids in phagosome/phagolysosome of macrophages. Macrophages that ingested GalCer purified from bovine spinal cord showed very similar flow cytomeric scatters (SSC^high^/FSC^low^ to SSC^high^/FSC^high^) and adipored-positive nature when compared to myelin-laden macrophages (Figures 2B,C).

Two subsets of CD1d-restricted NKT cells have been reported: namely invariant NKT (iNKT) cells which express invariant TCRα chain (Vα14-Jα18) paired with a limited number of TCRβ chains and noninvariant NKT cells which use a diverse TCR repertoire (22). iNKT cells, but not diverse NKT cells, are rapidly responsive to α-GalCer-loaded CD1d-multimers. To clarify the lipid antigen presented by myelin-laden macrophages, we first assessed myelin-laden macrophages using an antibody (clone L363) that specifically recognizes the α-GalCer:CD1d complex. We employed the resting macrophages (SSC^low^/FSC^low^) in same culture plate as a control to observe the immunofluorescence displayed by myelin-laden macrophages (SSC^high^/FSC^low^ to SSC^high^/FSC^high^). Surprisingly, immunofluorescence assays revealed positive presentations of α-GalCer via CD1d on myelin lipid-positive (adipored^high^) macrophages (Figure 2D, upper panel). Macrophages fed with GalCer purified from bovine spinal cords, which is mostly β-linked, showed α-GalCer presentation with similar efficiencies when compared with whole myelin-fed macrophages (Figure 2D, lower panel), suggesting that this antigenic presentation was mainly contributed by Mye-GalCer. Flow cytometric analysis confirmed that purified GalCer and whole myelin stimulated macrophages to present α-GalCer via CD1d to same extent (Figure 2E). However, unlike the whole myelin, purified GalCer triggered cell death of macrophages to some degree at later stage, and in turn different cytokine milieu (data not shown), presumably due to the cell death.

### Mye-GalCer Presentation-Restricted Cytokine Release

**Figure 3:**
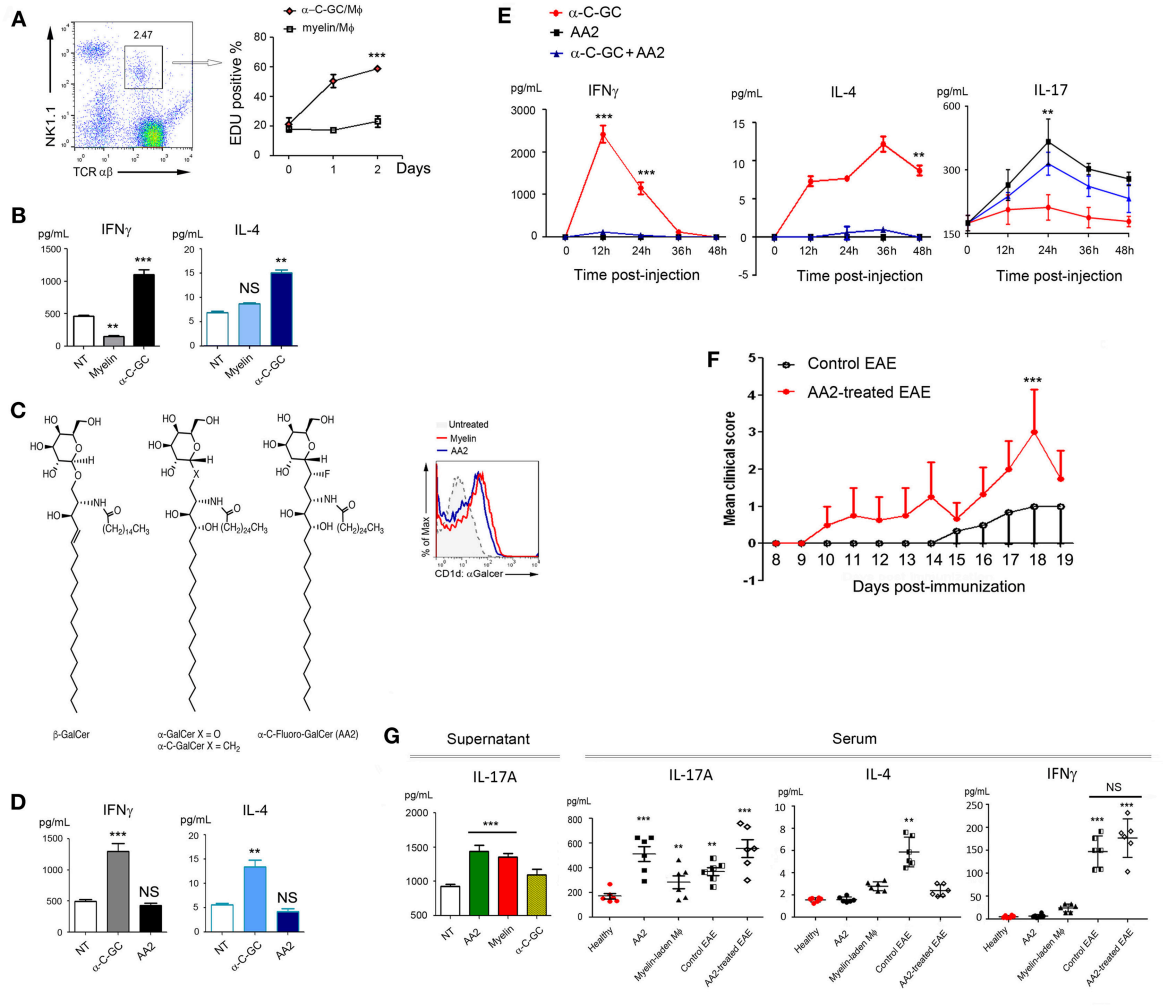
Elevated IL-17 expression following antigenic presentation of Mye-Galcer. **(A)** Gating strategies for NK1.1^+^/TCRαβ^+^ cells (left) and proliferation of NK1.1^+^/TCRαβ^+^ cells when co-cultured with myelin- or α-C-GC laden macrophages (right). Y-axis represents the percentage of EDU positive cells; X-axis presents time (day) post-EDU addition. **(B)** IFN_⋎_ and IL-4 expression in the co-culture system described in **(A)**. **(C)** Chemical structures of β-GalCer and α-C-Fluoro-GalCer (AA2) and antigenic Galcer presentation by macrophages treated with myelin debris or AA2 (CD1d::Galcer complex was detected by clone L363). **(D)** IFNγ and IL-4 expression in the co-culture system comprising splenetic T cells and α-C-GC- and AA2- treated macrophages. **(E)** Quantification of IFNγ, IL-4, and IL-17A in mouse sera collected from mice treated with α-C-GC, AA2, or the combination of α-C-GC and AA2 at indicated time points. **(F)** Disease progression of untreated and AA2-treated EAE mice. For evaluating the exacerbating effects of AA2, induction of EAE by MOG_35−55_ without PTX booster was employed. **(G)** Left: IL-17A expression in the co-culture systems comprising splenetic T cells and macrophages treated with AA2, myelin or α-C-GC; right: quantification of IL-17A in mouse sera collected from healthy mice treated with or without AA2, healthy mice adoptively transferred with myelin-laden macrophages, EAE mice and EAE mice treated AA2. IL-4 and IFNγ in same experiment setting were shown as controls. ^**^p < 0.05, and ^***^p < 0.01.

Both natural and synthetic α-GalCer are ligands for TCRs of iNKT cells (23, 24). Upon activation, iNKT cells proliferate and produce pro-inflammatory Th1 and anti-inflammatory Th2 cytokines. We therefore employed a co-culture system including myelin-laden macrophages and splenetic T cells to examine IFNγ and IL-4, the signature cytokines of iNKT cells (25, 26). To our surprise, myelin-laden macrophages, while presenting Mye-GalCer in α-form, stimulated neither expansion/proliferation of NK1.1^+^/TCRαβ^+^ cells (23) nor production of IFNγ and IL-4, in sharp contrast to synthetic α-C-GalCer in the co-culture system (Figures 3A,B).

Myelin is a complex coating that protects neuron fibers. In order to dissect the role of Mye-GalCer from other myelin components, studies with purified Mye-GalCer were desired. However, the unexpected cell death elicited by the purified bovine GalCer urged us to seek for a monomer mimicking Mye-GalCer in terms of all its subsequent bioactivities. Given that a subtle structural alteration of glycolipid ligand could largely affect the affinity of the glycolipid-CD1d complex to TCR of NKT cells, and in turn markedly different cytokine responses (16), we designed a series of modified α-C-GalCers and screened them in both co-culture system and C57/B6 mice. We discovered that α-C-Fluoro-GalCer (AA2) mimicked Mye-Galcer. As shown in Figure 3C, AA2 was efficiently presented via CD1d and readily detectable by antibody L363, while neither proliferation of NK1.1^+^/ TCRαβ^+^ cells (Figure S1) nor cytokine production (IFNγ & IL-4) was significantly stimulated *in vivo* and *in vitro* (Figures 3D,E, left and middle). Empowered with this Mye-Galcer-mimicking lipid monomer, we were able to study an IFNγ & IL-4-independent mechanism subsequent to glycolipid presentation. We observed that AA2 exacerbated the symptoms of MOG_33−55_-initiated EAE, as evidenced by earlier onset of disease and significantly higher EAE scores (Figure 3F). This interesting observation prompted us to ask which cytokine(s) other than IFNγ and IL-4 was responsive to AA2 challenge. The inflammatory cytokine IL-17 driven by both RORγt and T-bet has been shown to be crucial to autoimmune demyelinating diseases of CNS (27, 28). We, therefore, examined IL-17A production in response to AA2 challenge. As we expected, the serum level of IL-17A was dramatically elevated in AA2-treated healthy mice (Figure 3E right). We further employed the co-culture system including AA2-pulsed macrophages and splenetic T cells. Co-culture systems with myelin-laden- or α-C-Galcer-pulsed macrophages as previously described served as controls. Not surprisingly, the myelin-mimicking AA2 and myelin, but not α-C-Galcer, increased production of IL-17A in co-culture system (Figure 3G, left). We next tested sera collected from EAE mice treated with or without AA2. Sera collected from healthy mice received PBS, AA2, and adoptive-transferred, myelin-laden-macrophages served as controls. Consistently, as shown in Figure 3G (left), AA2 further elevated IL-17A levels, but not IL-4 and IFN_⋎_, in sera of EAE mice (Figure 3G, right).

**Figure 4:**
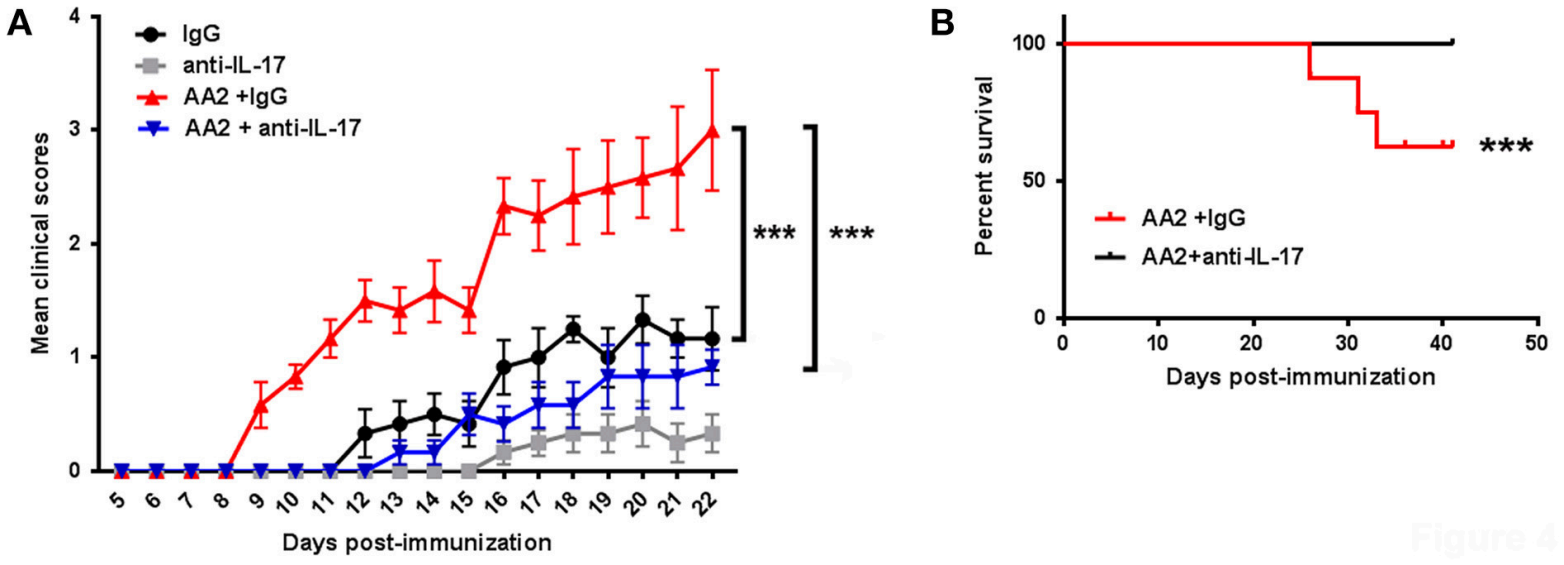
Role of IL-17 in AA2-exacerbated EAE. **(A)** Mice were induced with EAE and treated with IgG, IL-17 neutralizing antibody (anti-IL-17), AA2 combined with IgG, or AA2 combined with anti-IL-17 (*n* = 6 mice/group). The course of disease was monitored and clinical scores were determined. **(B)** Survival curves of EAE mice treated with AA2 combined with IgG or AA2 combined with anti-IL-17. There was no death in groups treated with IgG alone or anti-IL17 alone. For evaluating the exacerbating effects of AA2, induction of EAE by MOG_35−55_ without PTX booster was employed. ^***^p < 0.01.

Finally, to confirm that AA2-induced exacerbation of EAE symptoms was IL-17-mediated, AA2-treated EAE mice were co-treated with either IL-17A neutralizing antibody or its isotype control. As expected, IL-17A neutralizing antibody significantly ameliorated the EAE symptoms exacerbated by AA2 (Figures 4A,B and Videos S1, S2).

### Deprivation of Mye-Galcer Presentation by D-Sphingosine

**Figure 5:**
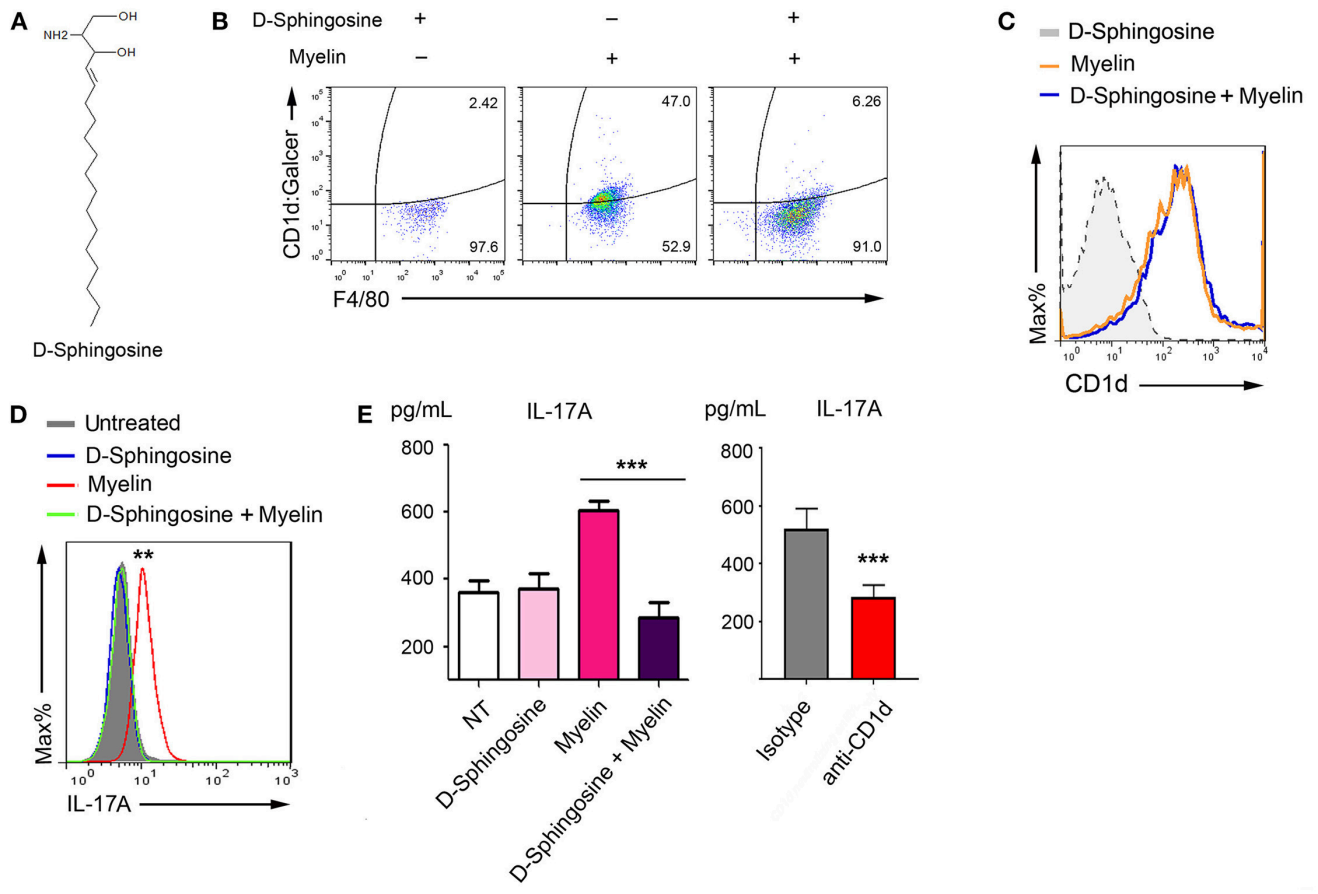
Deprivation of antigenic presentation of Mye-Galcer by D-Sphingosine. **(A)** Chemical sketch of D-Sphingosine. **(B)** Flow cytometric analysis of Mye-Galcer presentation by macrophages treated with D-Sphingosine, myelin, or the combination of D-Sphingosine and myelin. Macrophages were cultured in 10% cs-FBS media during the treatments. **(C)** Flow cytometric analysis of CD1d up-regulation by macrophages described in 4 **(B)**. **(D,E)** IL-17A production in myelin-laden macrophages/T cell co-culture system treated with or without D-sphingosine measured by CAB assays coupled with Flow cytometric analysis **(D)** and ELISA **(E)**. CD1d neutralizing antibody was employed as in myelin-laden macrophages/T cell co-culture system as an additional control to confirm that the increase of IL-17 is due to CD1d-mediated antigen presentation (**E** right). ^**^p < 0.05, and ^***^p < 0.01. Data are representative of 2–3 independent experiments.

Our findings that both myelin-originated and AA2-simulated α-GalCer presentation associated with the elevated IL-17 production raised the question whether abrogation of this glycolipid antigen presentation could impede IL-17 production. In this context, we next explored myelin lipids. The primary lipid of myelin is β-GalCer with a monogalactose glycosidically linked to the C-1 hydroxyl group of ceramide. Ceramide could be further broken down to sphingosine via ceramidase. Sphingosine (skeptical presentation shown in Figure 5A) and sphingosine-1-phosphate (S1P) are interchangeable via phosphorylation and dephosphorylation mediated, respectively, by sphingosine kinase type 1/2 and S1P phosphatase/S1P lysase (29, 30). However, S1P could not be efficiently formed in medium supplemented with 10% charcoal-stripped serum (cs-FBS) (31). We discovered that in medium supplemented with 10% cs-FBS, D-sphingosine (insoluble fine particles), but not S1P, efficiently blocked Mye-GalCer presentation on myelin-laden macrophages (Figure 5B). Treatment with D-sphingosine did not abolish the upregualtion of CD1d of myelin-laden macrophages, because when being stained with antibody that only recognizes CD1d protein, no difference was observed between D-sphingosine-treated and untreated myelin-laden macrophages (Figure 5C). These observations suggested that D-sphingosine only occupied the antigen-binding cleft without affecting CD1d expression.

To determine whether IL-17, the key cytokine associated with Mye-GalCer presentation, was also inhibited by D-sphingosine, we employed capture antibody-coated beads (CABs) (Figure 5D) and ELISA (Figure 5E) to test the IL-17A in the co-culture of myelin-laden macrophages and T cells pulsed with or without D-sphingosine. CD1d-neutralizing antibody served as a negative control. As we expected, myelin-laden macrophages failed to elevate the level of IL-17A when pretreated with D-sphingosine.

### Substantial Amelioration of EAE Severity by D-Sphingosine Treatment

We hypothesized that blockade of Mye-GalCer presentation could lead to a significant decrease in IL-17-mediated immune responses and thus amelioration of EAE. To test this hypothesis, MOG_35−55_-immunized mice were treated with D-sphingosine (insoluble fine particles) intraperitoneally (*i.p*.) or intravenously (*i.v*.).

**Figure 6:**
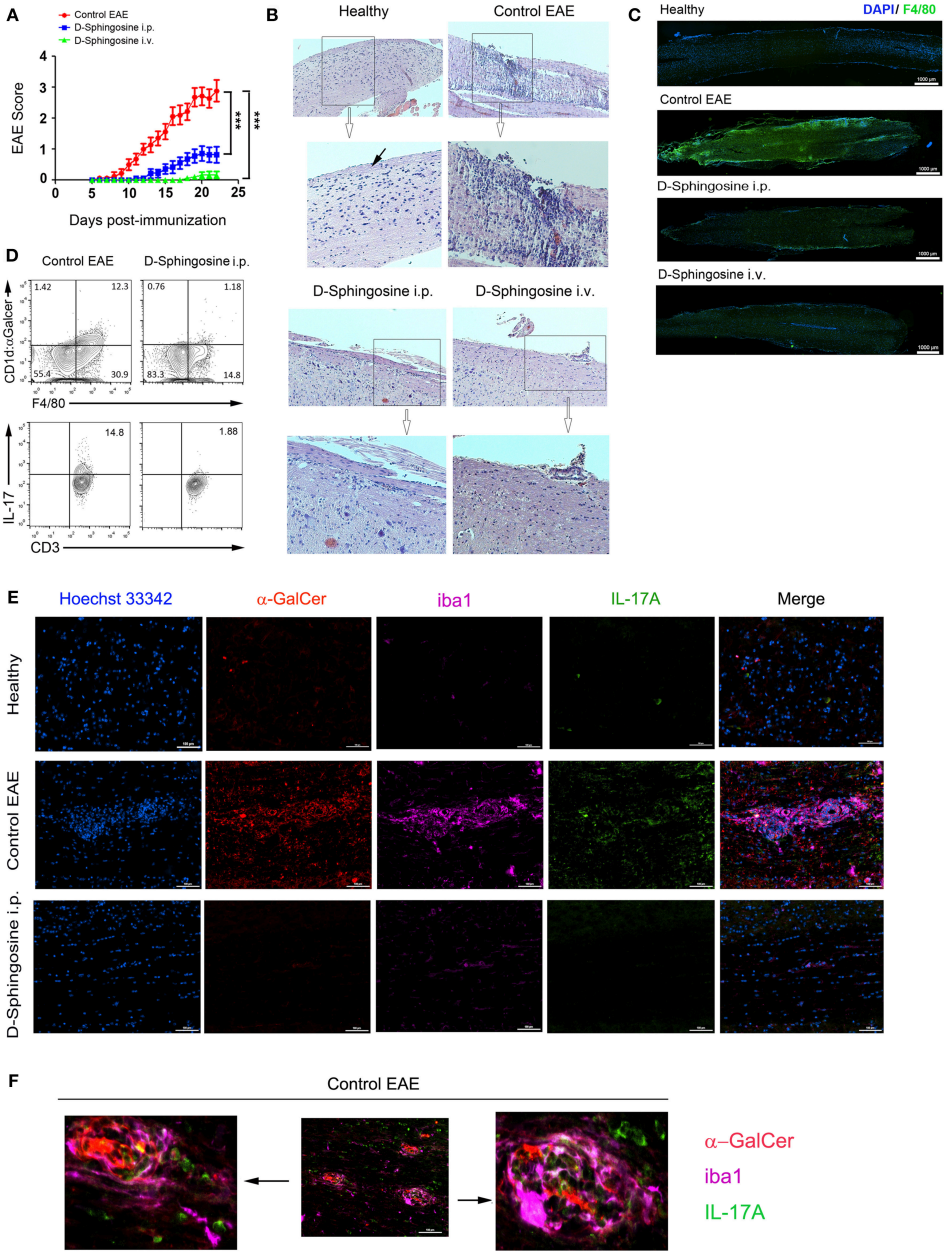
Therapeutic effects of D-Sphingosine on EAE. **(A)** Clinical scores and disease onsets of EAE mice treated with or without D-Sphingosine (*i.p* or *i.v*.). **(B)** H&E staining of spinal cords from healthy and the EAE mice described in 5 **(A)**. Pia matter indicated by a black arrow. Amplification 10x. **(C)** Fluorescent microscopy of the distribution of F4/80^+^ microglia/macrophages below lumbar segment L1 of spinal cords from healthy and the EAE mice described in **(A)**. **(D)** Flow cytometric analyses of Mye-Galcer presentation by microglia/macrophages and IL-17A production by T cells in spinal cords from EAE mice treated with or without D-Sphingosine (*i.p*.). **(E,F)** Fluorescent microscopy of CD1d:α-GalCer (red), microglia/macrophage clusters (iba1, magenta) and IL-17-producing cells (Green) in spinal cords from healthy and the MOG_35−55_-induced EAE mice treated with or without D-Sphingosine (*i.p*.) (*n* = 5/group). ^***^p < 0.01. Data are representative of 3 independent experiments.

Both *i.p*. and *i.v*. groups of mice receiving D-Sphingosine demonstrated significantly lowered clinical scores and delayed onsets compared to control EAE mice. The mean scores of *i.v*. group were further lower by a grade of 0.61 ± 0.11 than i.p. group (Figure 6A, Supplementary Table 1, Videos S3, S4). Histopathologically, control mice with EAE displayed extensive necrotizing lesions of pia matter and subpial inflammatory infiltrates; in contrast, D-Sphingosine-treated mice at the same time points showed dramatically more restricted pia matter necrosis and striking reductions of subpial inflammation [hematoxylin & eosin (H&E)-stained spinal cord sections shown in Figure 6B]. Consistently, animals in *i.v*. group demonstrated even less impairment of spinal cord than those in *i.p*. group. It has been well-accepted that the demyelinating lesions are characterized and defined by both residing and infiltrating macrophages (19, 32–34). We next employed the microglia/macrophage marker F4/80 to examine the spinal cord portion below lumbar segment L1. As shown in Figure 6C, F4/80^+^ cells significantly concentrated throughout lumbar to sacral segments in control EAE mice; whereas such a clustering of macrophages dissipated upon D-sphingosine treatment regardless of the administration routes. Depletion of hematogenous macrophages has been shown to promote myelin preservation and neuron sprouting (35). Thus, our observations of D-sphingosine-induced reduction of macrophages paralleled with its therapeutic effects in EAE mice. More importantly, flow cytometric analysis showed that the Mye-GalCer presentation by macrophages *in situ* of EAE spinal cord was notably abrogated by D-sphingosine treatment (Figure 6D, upper). In the same experimental settings, the expression of cytokine IL-17 of CD3^+^ population was investigated. A strong correlation between myelin-originated Mye-GalCer presentation by macrophages and production of IL-17A by CD3^+^ cells in spinal cords was observed (Figure 6D, lower). We further discovered that IL-17A^+^ T cells in control EAE and AA2-treated EAE spinal cords were largely CD3^+^/NK1.1^−^/CD4^−^/CD8^+/−^, suggesting that the downstream effector T cells were not the canonical iNKT cells (Figure S2).

We next set out to determine the micro-locations of the lipid antigen and IL-17A via immunofluorescence analysis of EAE spinal cords with or without D-sphingosine treatment. As shown in Figure 6E (upper and middle panels), the spinal cord from healthy mice was largely devoid of macrophages clusters, which were identified by microglia/macrophage-specific protein, ionized calcium binding adapter molecule 1 [iba1, (36)]. Neither Mye-GalCer presentation (red) nor IL-17A expression (green) was markedly evident in healthy spinal cord. However, the spinal cord of control EAE mice exhibited multiple macrophage plaques presenting α-GalCer. IL-17A-producing cells were found partially co-localizing with macrophage plaques. Direct conjugation of IL-17A-producing cells to GalCer antigen was also observed (Figure 6F). In contrast, D-sphingosine abrogated myelin-originated GalCer presentation by macrophages, mitigated the macrophage plaques and the accumulation of IL-17A-producing cells on or near the macrophage plaques (Figure 6E, lower panel). Collectively, our study demonstrated an axis from lipid antigen presentation to IL-17 production.

## Discussion

CD1d-restricted NKT cells have been shown to possess abilities to produce diverse cytokines, including IFNγ, IL-2, IL-3, IL-4, IL-6, IL-10, IL-17, etc. Compared to other NKT subsets, an unconventional CD4^−^/NK1.1^−^ NKT cell population appears to be primarily responsible for the production of IL-17 (referred as NKT-17 hereafter) (13). Consistent with the previous findings, we discovered that the IL-17-producing T cells activated by endogenous mye-Galcer or AA2 are largely CD3^**+**^/ CD4^**−**^/NK1.1^−^/CD8^+/−^ (Figure S2). It is nevertheless unclear precisely how the “on-switch” of NKT-17 is triggered in the autoimmune disease settings. On the other hand, myelin-phagocytosing macrophages (foamy macrophages) was viewed as anti-inflammatory regulators with an important role of clearing myelin debris as a prerequisite for remyelination (32, 37–40). Conversely, the pro-inflammatory characteristics of macrophages after being exposed to myelin debris *in vitro* and *in vivo* have also been reported (19, 41–44). While partially consistent with anti-inflammatory role in regard to low IFNγ/IL-4 ratio, our findings point to the possibility that after myelin debris internalization, α-GalCer-presenting macrophages lead to the increased production of IL-17. This observation was corroborated by the previous reports that in inflamed spinal cord, foamy macrophages strongly express IL-6, the cytokine essential for encephalitogenic IL-17^+^ cell differentiation (28, 32, 45). We demonstrated for the first time that the myelin-laden macrophage serves as a pro-inflammatory player in a non-canonical way in EAE spinal cords, and shed a considerable light for reconciling the detrimental role of myelin-laden macrophages with its incapability of eliciting IFNγ. Our data showed that NK1.1^+^ iNKT were not reactive when co-cultured with myelin-laden macrophages, further investigation are needed to confirm that the NKT-17 cells are the downstream effectors responding to the myelin-derived, α-GalCer presentation.

**Figure 7:**
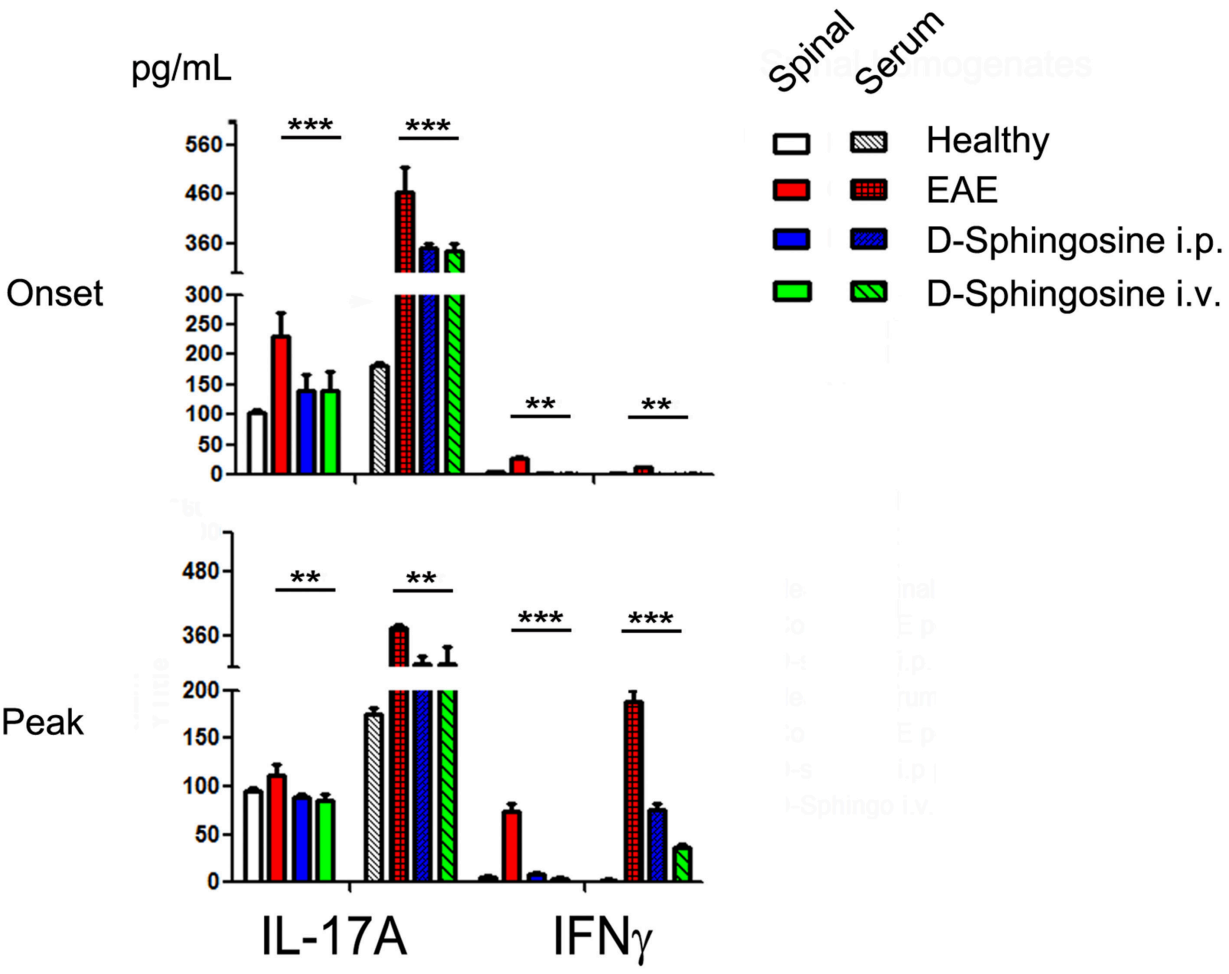
Phased cytokine production in EAE mice. Quantification of IL-17 and IFN_⋎_ in sera and spinal cord homogenates collected from EAE mice treated with or without D-Sphingosine (*i.p*. or *i.v*.) at the onset and the peak of disease (*n* = 6/group). ^**^p < 0.05, and ^***^p < 0.01.

Both Th1 and Th-17 cells have been proposed to be involved in pathogenesis of EAE (27). Observations that IFNγ-deficient mice are susceptible to EAE (46), and IL-17-deficient mice still developed EAE only with less severe symptoms (47, 48) indicate that induction and progression of EAE are dependent on an imbalanced cytokine network, not a single pro-inflammatory mediator. Our investigations on supernatants of spinal cord homogenates and sera collected from EAE mice showed that IL-17A, not IFNγ, is one of the predominant cytokines at the disease onset stage locally (spinal cord) and systematically (serum); quenching this early expression of IL-17A by D-sphingosine decreased IFNγ in spinal cord at the peak of disease (Figure 7). IL-1β and TNFα also declined to various degrees in D-sphingosine-treated spinal cords (data not shown). Together, our findings suggest Mye-GalCer presentation-associated IL-17A expression at the onset stage of EAE plays an important role in building up the inflammatory network at disease climax, and highlight the therapeutic potentials of D-sphingosine.

Intravenous administration of D-sphingosine could involve the effects of both sphingosine and S1P resulted from phosphorylation of sphingosine in blood stream, since the signaling axis of ApoM-S1P-S1P receptor 1 significantly restrains lymphopoiesis, and in turn the adaptive immune responses in CNS (49). Intraperitoneal administration of insoluble fine particles (D-sphingosine resuspended in PBS), in contrast, are known to be absorbed to lymphatic circulation (50, 51) where most of D-sphingosine are likely to stay in an unphosphorylated form before they are engulfed by phagocytes, due to the much lower level of ApoM in lymph than in blood (49, 52). These explain why D-sphingosine *i.v*. achieved better clinical cores than *i.p*. group while having comparable efficiency of abrogating the lipid antigen presentation.

## Author Contributions

YB, YR, and XM designed the studies. YB, WD, LZ, and TZ performed biology experiments. AA, KA, and DM synthesized all the glycolipids. VB and TH provide EAE mouse model and supervised animal experiments. YB was the major contributors in writing the manuscript. XM and DM revised the manuscript. All authors read and approved the final manuscript.

### Conflict of Interest Statement

The authors declare that the research was conducted in the absence of any commercial or financial relationships that could be construed as a potential conflict of interest.

## Supplementary Material

Videos S1 EAE mice treated with AA2 and IgG (Isotype control) at Day 9.

Videos S2 EAE mice treated with AA2 and anti-IL-17 antibody at Day 9.

Videos S3 EAE mice treated with Vehicle (control EAE) at Day 21.

Videos S4 EAE mice treated with D-sphingosine (i.p.) at Day 21.
