# Supplementary material for: Abrogation of Endogenous Glycolipid Antigen Presentation on Myelin-Laden Macrophages by D-Sphingosine Ameliorates the Pathogenesis of Experimental Autoimmune Encephalomyelitis
Source: Front Immunol. 2019 Mar 19;10:404. doi: 10.3389/fimmu.2019.00404 (PMC6433838; doi:10.3389/fimmu.2019.00404)
Supplement: Supplementary file 5 [file Table_1.pdf]

Tabel 1.
Detailed clinical EAE development of PBS-treated (Control EAE) *vs.* D-Sphingosine-treated mice.

	Day of onset [a]	Incidence	Maximum score [a]
Control EAE	10.3 ± 0.88	93% (13/14)	4.17 ± 0.44
D-Sphingosine i.p.	13.6 ± 0.57	29% (4/14)	2.26 ± 0.37
D-Sphingosine i.v.	17.3 ± 0.88	13% (2/15)	1.33 ± 0.17

[a]: Mean of the diseased mice (Mean ± SEM)
