# Supplementary material for: Abrogation of Endogenous Glycolipid Antigen Presentation on Myelin-Laden Macrophages by D-Sphingosine Ameliorates the Pathogenesis of Experimental Autoimmune Encephalomyelitis
Source: Front Immunol. 2019 Mar 19;10:404. doi: 10.3389/fimmu.2019.00404 (PMC6433838; doi:10.3389/fimmu.2019.00404)
Supplement: Supplementary file 6 [file Data_Sheet_1.PDF]

Supplementary Figures for

Abrogation of Endogenous Glycolipid Antigen Presentation on Myelin-laden Macrophages by D-Sphingosine Ameliorates the Pathogenesis of Experimental Autoimmune Encephalomyelitis

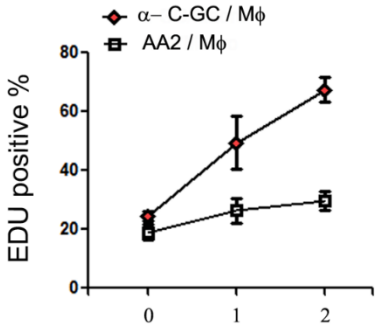

Figure S1. Proliferation of NK1.1^{+}/ TCRαβ^{+} cells in response to AA2 challenge. In a co-culture system comprising splenic T cells and macrophages treated with α-C-GC or AA2, EDU incorporation by NK1.1^{+}/ TCRαβ^{+} cells was measured via flow cytometry.

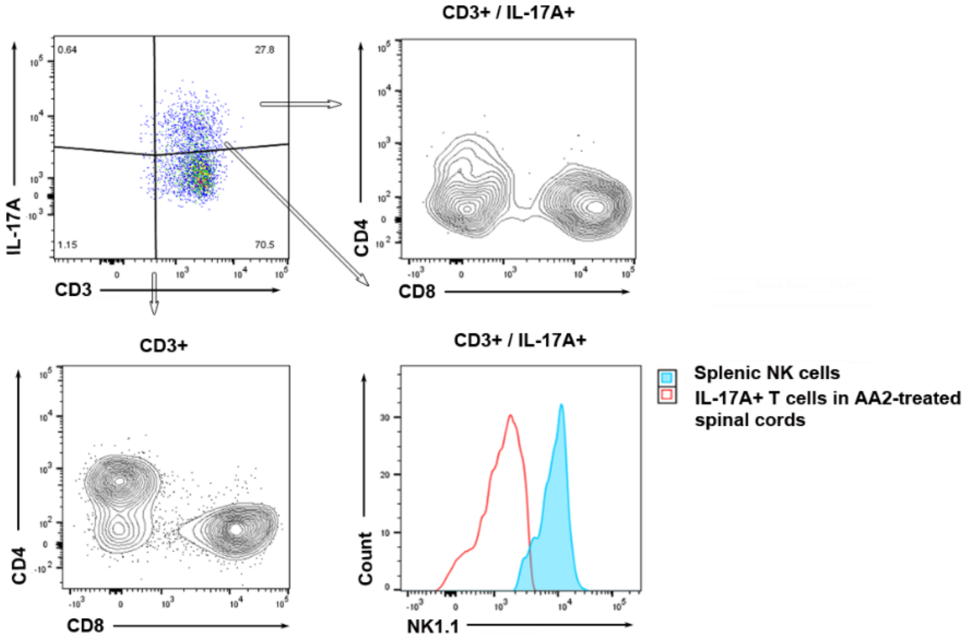

Figure S2: Characterization of IL-17 producing T cells in response to glycolipid antigen. Representative flow cytometric results showing that in spinal cords of AA2-treated EAE mices, IL-17A positive cells were largely CD3^{+}/ NK1.1^{-} / CD4^{-}/CD8$^{+/-}$.
